# Spatial distribution and risk factors of Brucellosis in Iberian wild ungulates

**DOI:** 10.1186/1471-2334-10-46

**Published:** 2010-03-05

**Authors:** Pilar M Muñoz, Mariana Boadella, Maricruz Arnal, María J de Miguel, Miguel Revilla, David Martínez, Joaquín Vicente, Pelayo Acevedo, Álvaro Oleaga, Francisco Ruiz-Fons, Clara M Marín, José M Prieto, José de la Fuente, Marta Barral, Montserrat Barberán, Daniel Fernández de Luco, José M Blasco, Christian Gortázar

**Affiliations:** 1 Centro de Investigación y Tecnología Agroalimentaria del Gobierno de Aragón (CITA). Montañana, 930 50059, Zaragoza. Spain; 2 Instituto de Agrobiotecnología CSIC-UPNA-Gobierno de Navarra, 31192 Mutilva Baja, Spain; 3 IREC (CSIC-UCLM-JCCM). Ronda de Toledo s/n, 13071 Ciudad Real, Spain; 4 Departamento de Patología Animal de la Universidad de Zaragoza. Miguel Servet, 177 50013, Zaragoza, Spain; 5 Biogeography, Diversity, and Conservation Research Team, Animal Biology, Department of Sciences, University of Malaga, E-29071 Málaga, Spain; 6 SERIDA, Servicio Regional de Investigación y Desarrollo Agroalimentario, Laboratorio de Sanidad Animal, 33299 Jove, Gijón, Spain; 7 NEIKER-TECNALIA, Inst Vasco Invest & Desarrollo Agrario, Dpt Anim Hlth, Bizkaia 48160, Spain; 8 Department of Veterinary Pathobiology, Center for Veterinary Health Sciences, Oklahoma State University, Stillwater, OK 74078, USA

## Abstract

**Background:**

The role of wildlife as a brucellosis reservoir for humans and domestic livestock remains to be properly established. The aim of this work was to determine the aetiology, apparent prevalence, spatial distribution and risk factors for brucellosis transmission in several Iberian wild ungulates.

**Methods:**

A multi-species indirect immunosorbent assay (iELISA) using *Brucella *S-LPS antigen was developed. In several regions having brucellosis in livestock, individual serum samples were taken between 1999 and 2009 from 2,579 wild bovids, 6,448 wild cervids and4,454 Eurasian wild boar (*Sus scrofa*), and tested to assess brucellosis apparent prevalence. Strains isolated from wild boar were characterized to identify the presence of markers shared with the strains isolated from domestic pigs.

**Results:**

Mean apparent prevalence below 0.5% was identified in chamois (*Rupicapra pyrenaica*), Iberian wild goat (*Capra pyrenaica*), and red deer (*Cervus elaphus*). Roe deer (*Capreolus capreolus*), fallow deer (*Dama dama*), mouflon (*Ovis aries*) and Barbary sheep (*Ammotragus lervia*) tested were seronegative. Only one red deer and one Iberian wild goat resulted positive in culture, isolating *B. abortus *biovar 1 and *B. melitensis *biovar 1, respectively. Apparent prevalence in wild boar ranged from 25% to 46% in the different regions studied, with the highest figures detected in South-Central Spain. The probability of wild boar being positive in the iELISA was also affected by age, age-by-sex interaction, sampling month, and the density of outdoor domestic pigs. A total of 104 bacterial isolates were obtained from wild boar, being all identified as *B. suis *biovar 2. DNA polymorphisms were similar to those found in domestic pigs.

**Conclusions:**

In conclusion, brucellosis in wild boar is widespread in the Iberian Peninsula, thus representing an important threat for domestic pigs. By contrast, wild ruminants were not identified as a significant brucellosis reservoir for livestock.

## Background

Brucellosis is an infectious disease caused by bacteria of the genus *Brucella*, characterized by abortion and infertility in several mammal species, and being considered one of the most important zoonosis worldwide [1]. *Brucella melitensis*, followed by *Brucella abortus *and *Brucella suis*, are the main species involved in the infection of human beings, thus being the main target of eradication campaigns.

With very few exceptions, *B. suis *infection in both humans and pigs remains an important problem in most countries. *B. suis *biovar 2 is the main responsible of brucellosis in pigs in Europe. Despite having been isolated from human beings [2], this biovar 2 seems to be less pathogenic for humans than the biovars 1 and 3 [3]. Other *Brucella *species have been isolated in rodents, terrestrial carnivores, and sea mammals, but the relevance of these *Brucella *species for livestock and human beings is quite limited [3-5].

Wild animals are often at risk as a consequence of contacts with infected livestock, particularly in extensive breeding systems. In addition to the *B. abortus *infection specific problem shared by cattle, bison (*Bison bison*) and elk (*Cervus elaphus*) in limited territories of the USA (see below), some sporadic cases have been reported in wild bovids, such as ibex (*Capra ibex*) and chamois (*Rupicapra *sp.) in the EU [6,7]. Although wild ruminants have been suggested to hold brucellosis and eventually originate spillback to domestic animals or infection in humans, the most extended opinion is that these wild animals are occasional victims of brucellosis transmitted from infected livestock, rather than a true reservoir of the disease for domestic animals [8]. In fact, only limited cases of brucellosis have been reported in these free-living animals [3,9,10], and only weak evidence for a direct relationship between brucellosis apparent prevalence and wild ruminant population size/density has been found (e.g. [11] and references therein). However, the risk can be high in overabundant wildlife populations in contact with infected livestock and when artificial management increases aggregation [8,11]. In the Greater Yellowstone Ecosystem in the USA, winter feeding of elk and bison contributes to maintain valuable wildlife populations and avoid contacts between *B. abortus *infected wildlife and cattle, but significantly increases the intra-specific transmission risk [12]. Modelling of observational data has shown that brucellosis prevalence in elk correlates with the timing of the winter feeding season [13]. This underlines that human dimension issues are fundamental to successful management of wildlife diseases [11].

Brucellosis caused by *B. suis *biovar 2 is frequently reported in the Eurasian wild boar (*Sus scrofa*) and the European brown hare (*Lepus europaeus*), and apparent prevalence ranging from 8 to 32% has been reported in wild boar in the EU [10,14-18]. It is accepted that both species play a relevant role as a brucellosis reservoir for domestic pigs, even under natural environmental conditions [3,15,19]. In fact, both wildlife species have been directly involved in the transmission of infection to domestic pigs reared in outdoor farms [10]. Outside the EU, feral pigs may maintain *B. suis *biovars 1 and 3, being a potential source of infection to both domestic pigs and human beings [20].

Only limited information on wildlife brucellosis is available in the Iberian Peninsula. Regarding wild ruminants, brucellosis has not been detected in limited studies conducted on Barbary sheep (*Ammotragus lervia*) [21], Cantabrian chamois (*Rupicapra pyrenaica parva*) [22] and mouflon (*Ovis aries*) [23]. In contrast, several cases of infections induced by *B. suis *biovar 2, have been reported in wild boar [24] and European brown hares [25]. Wild ungulates are currently expanding and increasing in density in the whole Iberian Peninsula [26], as well as the artificial management of these wild species including fencing, feeding and translocation, then increasing the risk of infectious disease transmission [27].

The availability of accurate and validated diagnostic tests is of paramount importance to properly assessing the prevalence of brucellosis in wildlife [28]. In this work we developed a multispecies iELISA to determine brucellosis apparent prevalence in several Iberian wild ungulate species, and determined spatial distribution and risk factors associated with brucellosis. We hypothesised that: (1) free-living wild ruminants would not show significant infection with *Brucella *species; (2) wild boar, conversely, would show infection with *B. suis *biovar 2, constituting a potential hazard for domestic pigs; and (3) apparent prevalence would vary with environmental, population and individual risk factors such as artificial management.

## Methods

### Study area

The study area was the Iberian Peninsula in the south-western European Union. This includes a variety of habitats and climates, which can be simplified into 5 different Bio-regions in the mainland, as defined in the Spanish Wildlife Disease Surveillance Scheme (Internal report to the Spanish Ministry of Agriculture, MARM and spatial aggregation of wildlife. 2008). Table 1 summarises the most relevant characteristics of each Bio-region.

**Table 1:** Characteristics of the Bio-regions of the Iberian Peninsula included in the study area

Bio-region	Environment	Wildlife	Sampling site characteristics
1.- Atlantic	Atlantic climate with high precipitation. Pastures and deciduous woodlands. Mountain habitats. Almost no fencing of wildlife habitats.	Wild boar and roe deer abundant. Locally red deer abundant. Chamois at high altitudes (Cantabrian Mts.).	N = 76. Woodlands: 62%; Agricultural lands: 33%. Altitude (in m): mean 452 (range 0-2032). Mean annual precipitations (in mm): 1284. Mean annual temperature (in °C): 12
2.- Northern-Plateau	Continental Mediterranean climate. Dry, hot summers, dry, cold winters. Open, cereal landscapes with pine or oak woodlands, limited to the north by mountains. Little fencing.	Ungulates expanding and locally abundant. Chamois limited to high altitudes in the Pyrenees. Locally ibex and fallow deer.	N = 98. Woodlands: 68%; Agricultural lands: 30%. Altitude (in m): mean 987 (range 67-3314). Mean annual precipitations (in mm): 808. Mean annual temperature (in °C): 10.5
3.- South-Central	Continental Thermo Mediterranean climate. Pastures and crops with interspersed vegetation, sometimes forming savannah-like structures. Low altitude mountains with scrubland. Frequent fencing.	Wild boar and red deer often at high density; feeding and watering. Locally abundant fallow deer and Iberian ibex, and introduced wild bovids.	N = 72. Woodlands: 68%; Agricultural lands: 29%. Altitude (in m): mean 705 (range 47-2321). Mean annual precipitations (in mm): 605. Mean annual temperature (in °C): 14.5
4.-Interior Mountains	Severe Continental Mediterranean climate. Limestone mountain and high-plateau habitats with cereal crops, pastures, and pine and oak woodlands. Little fencing.	Wild boar, roe deer, and ibex widely distributed but usually at moderate abundance. Locally abundant red deer.	N = 22. Woodlands: 71%; Agricultural lands: 29%. Altitude (in m): mean 1178 (range 248-1932). Mean annual precipitations (in mm): 568. Mean annual temperature (in °C): 11.3
5.- South and East Coast	Coastal Thermo Mediterranean climate; arid in the central portion. Few well preserved wildlife habitats (mountains). Little fencing.	Wild boar abundant in the northern and southern ends. Other ungulates locally abundant.	N = 7. Woodlands: 48%; Agricultural lands: 23%. Altitude (in m): mean 190 (range 0-1238). Mean annual precipitations (in mm): 720. Mean annual temperature (in °C): 15.7

### Animal sampling procedures

The number of samples obtained by species and study region is summarised in Table 2. Sampling was opportunistic and biased towards the hunting season (October to February in most species, and summer in chamois and roe deer), and took place from 1999/2000 to 2008/2009. The total number of wild ungulates sampled was 13,481, including 2,579 bovids (Barbary sheep, chamois, Iberian wild goat and mouflon), 6,448 cervids (roe deer, red deer and fallow deer) - see Table 2 for the precise numbers in each animal species-, and 4,454 wild boar. Samples were collected from hunter-harvested animals. Blood was drawn from the heart or the thoracic cavity during field necropsies, then the serum (usually haemolysed) was collected after centrifugation and kept frozen at -20°C until analysed. Whenever possible, cranial and iliac lymph nodes, spleen and sexual organs were collected and stored at -20°C for microbiological analyses. The number of samples from the different animal species submitted to microbiological studies is shown in Table 2.

**Table 2:** Sample size by host species and Bio-region studied, apparent prevalence obtained, and *Brucella *culture results in Iberian wild ungulate species.

Common name	Latin name	Serum samples by region						Mean prevalence (95% CI)	Samples submitted for culture	Nr. of isolates (species and biovar)
					
		1	2	3	4	5	Total			
**Barbary sheep**	*Ammotragus lervia*	0	0	8	0	0	8	0 (0-36)	0	
**Mouflon**	*Ovis aries*	0	0	75	0	0	75	0 (0-5)	0	
**Iberian wild goat^1^**	*Capra pyrenaica*	0	41	2	1042	1	1086	0.1 (0-0.6)	1^2^	1 (*B. melitensis *biovar 1)
**Chamois^3^**	*Rupicapra pyrenaica*	57	1353	0	0	0	1410	0.8 (0.4-1.4)	11	
**Roe deer**	*Capreolus capreolus*	77	152	5	9	42	285	0 (0-1)	0	
**Fallow deer**	*Dama dama*	92	107	47	32	64	342	0 (0-1)	0	
**Red deer**	*Cervus elaphus*	452	1591	2378	932	468	5821	0.4 (0.3-0.6)	81^4^	1 (*B. abortus *biovar 1)
**Wild boar**	*Sus scrofa*	658	1920	1499	132	245	4454	33 (31.6-34.4)	589^5^	104 (*B. suis *biovar 2)
**TOTAL**		1336	5164	4014	2147	820	13481		682	106

^1 ^Includes mainly the Mediterranean subspecies *Capra pyrenaica hispanica*. ^2 ^All animals were sampled randomly during hunting or at game farms but for the ibex tissues submitted for culture, which came from a clinical case with suspected brucellosis. ^3 ^Cantabrian chamois (*Rupicapra pyrenaica parva*) in Bio-region 1 and Pyrenean chamois (*R. p. pyrenaica*) in Bio-region 2. ^4 ^Thirty-one out of these 81 samples came from iELISA-positive animals and 50 from iELISA-negative ones. ^5 ^A total of 539 out of these 589 samples were from iELISA-positive animals and 50 from iELISA-negative ones.

Age-classes of biological meaning were defined. Based on tooth eruption patterns, wild ruminants were classified as fawns (first year of life), yearlings (second year of life), juveniles (third to fourth year of life), and adults (fifth year of life onwards). Wild boar less than 7 months old were classified as piglets, between 7 and 12 months were classified as juveniles, those between 12 and 24 months as sub-adults, and those over 2 years as adults [29]. Sex was known in 5,683 wild ruminants, and age-classes in 4,065. For wild boar, sex was known in 2,688 animals and age in 2,419.

### Serological studies

A multi-species indirect enzyme immunoassay (iELISA) was developed and validated to assess brucellosis apparent prevalence. Briefly, a phenol-water smooth lipopolysaccharide (S-LPS) rich extract from *B. melitensis *16M was obtained as described elsewhere [30]. Standard 96-well polystyrene plates (Maxisorp Nunc A/S, Roskilde, Denmark) were coated with 100 μl of an antigen solution (2.5 μg/mL) in phosphate-buffered saline (PBS; 10 mM, pH 7.2), and the plates incubated at 4°C overnight. After three consecutive washes with 0.05% Tween-PBS, the plates were ready for use. Then, 100 μl of the optimal dilution of each serum were added by duplicate to each well, and the plates incubated for 45 min at 37°C. Optimal serum dilutions (assessed using 20 sera from culture positive and 20 sera from *Brucella *free animals belonging the different domestic animal species used as controls -see below-) in 0.05% Tween-PBS were 1/100 (goats and phylogenetically related species) or 1/50 (the remaining animal species tested). The non-reacting antibodies were removed by three consecutive washes with 0.05% Tween-PBS. Then, a conjugate solution containing 0.2 μg/mL of recombinant protein G/HRP (Pierce Chemical Co., Rockford, Ill, USA) in 0.05% Tween-PBS was added (100 μl/well), and the plates incubated again for 45 min at 37°C. After three consecutive washes with 0.05% Tween-PBS to remove unbound conjugate, the reaction was developed with 100 μl/well of a 0.1% solution of 2,2-azinobis, 3-ethylbenzothiazoline sulfonic acid, diammonium salt (ABTS; Sigma Chemical Co., St. Louis, Mo., USA) and 0.004% hydrogen peroxide in 0.05 M citrate buffer (pH 4). The reaction was not stopped, and the OD at 405 nm was automatically assessed (Multiskan RC; Thermo Labsystems, Vantaa, Finland) after 15 min of incubation at room temperature in the dark. Results were expressed as the percentage of optical density (%OD) using the formula [% OD = 100 × mean OD of duplicated sample/mean OD of duplicate positive control]. Due to the lack of gold standard sera (*i.e.*, taken from culture positive and brucellosis free animals) from the different wild ungulate species, the sera used for setting up and iELISA validation were from *Brucella *culture positive (CP) and *Brucella*-free (BF) phylogenetically related domestic animals. Cattle sera were used as reference for red, roe and fallow deer; goat sera for chamois and Iberian wild goat; sheep sera for mouflon and Barbary sheep; and pig sera for wild boar. All gold standard sera from domestic species were available at the serum collection of the CITA (Zaragoza, Spain). To establish the optimal test conditions (*i.e.*, those allowing the maximum of separation of %OD values between the infected and free populations) for each animal species, sera from 88 CP and 88 BF cattle, 88 CP and 88 BF sheep, 44 CP and 88 BF goats and 62 CP and 100 BF pigs were used. The overall results were then submitted to ROC analyses (Medcalc. 9.2.1.0 software) and cut-offs resulting in 100% diagnostic specificity and the maximal diagnostic sensitivity for sheep, goats and cattle (50%OD), and pigs (40%OD), were selected to further assess the apparent prevalence in the corresponding phylogenetically wild animals tested.

### Bacteriological analysis and *Brucella* typing

Necropsy samples (lymph nodes, spleen and/or sexual organs) from iELISA-positive animals (see Table 2 for precise numbers in each species) were submitted to bacteriological analysis. To assess the relative diagnostic specificity of the iELISA developed, similar necropsy samples taken from iELISA-negative animals (see Table 2) were also cultured. Briefly, each sample was surface decontaminated by immersion in ethanol and gentle burning, introduced in sterile bags, suspended in the minimal amount of sterile PBS required for adequate homogenisation, and then homogenised in a blender (Stomacher; Seward Medical, London, UK). Each homogenate was smeared onto at least two plates of both Farrell's and modified Thayer Martin's culture media [31]. After 5-7 days of incubation at 37°C in 10% CO_2 _atmosphere, the resulting *Brucella *isolates were identified according to standard procedures [32].

*Brucella *field isolates were further analysed using both molecular and standard bacteriological procedures. Bacterial DNA was extracted using QIAamp DNA minikit (QIAGEN, Hamburg, Germany). For the identification and differentiation of *Brucella *species, the Bruce-ladder multiplex PCR was applied as described elsewhere [33]. To assess the precise biovar and the different haplotypes of *B. suis *biovar 2 strains isolated, a multiplex PCR [34] and PCR-RFLP of *omp31*, *omp2a *and *omp2b *genes [35,36] were used. The corresponding biovars of the two *B. melitensis *and *B. abortus *strains isolated were identified by agglutination with monospecific A and M antisera, and growth patterns in culture media containing Thionine and Basic Fuchsin (20 μg/ml) after incubation with and without CO_2 _atmospheres [32].

### Statistical analyses

We used Sterne's exact method (up to N = 1,000), or adjusted Wald method (N > 1,000) to estimate apparent prevalence confidence intervals [37]. Apparent prevalence comparisons among categories were done with homogeneity tests. The Mantel test was used to assess the spatial association between brucellosis apparent prevalence in wild boar across different sampling sites. Calculations were done with the PASSAGE software [38].

Quantitative exploratory analysis of risk factors for brucellosis apparent prevalence was carried out at two different geographic scales (peninsular and regional) using two-stage analyses. First, the associations between all the hypothesized risk factors and apparent prevalence were analyzed using single factor generalized models. Factors that captured the effect of any set of highly correlated variables for which P < 0.1 were selected for inclusion in the multivariate models (Table 3). In a second step, the selected variables were then jointly evaluated in a multiple logistic model. The individual iELISA result (N = 3,883) was the response variable (binomial, *i.e. *antibody presence or absence). Since sampling across different populations was not homogeneous in relation to age and sex, statistical analyses were conducted at the individual level to control for them. Age was included as a continuous discrete explanatory variable and sex was included as a categorical binomial explanatory variable. We used a stepwise strategy to obtain the final model. Statistical significance was assumed wherever P < 0.05. We used the SAS statistical package.

**Table 3:** Factors included in the analysis, indicating those significantly associated (excluding other highly correlated variables) with apparent prevalence of brucellosis at the Peninsular (GLZ, P < 0.1, N > 2416) and the regional (GLZ, P < 0.1, N > 460) scales.

Peninsular scale			
**Factor**	**Estimate**	**N**	**P**

Significantly associated with prevalence (selected for the model):			
Age class (1-4)		2416	< .0001
Month (1-12)		4394	< .0001
Annual rainfall	-0.00013	4079	0.0011
Cultivated lands	0.000629	4079	0.0091
Non-irrigated cultures	0.000908	4079	0.0181
Iberian hare habitat suitability	0.000011	4019	0.0287
Road	0.07015	4079	0.0386
Woodlands	(-0.000644)	4079	0.0529
Irrigated cultures	0.001514	4079	0.0709
Urban	0.00572	4079	0.0745
Not associated with prevalence (not selected):			
Sex (1-2), wild boar management, European brown hare habitat suitability, irrigated fruit orchards, pastures, annual radiation, slope range, mean slope, maximum slope, mean altitude, min. altitude, max. altitude, altitude range, annual temperature [69], annual temp. (min), annual temp. (max)			

**Regional scale**			

**Factor**	**Estimate:**	**N**	**p**

Selected:			
Age class			0.0001
Month (1-12)		505	0.0263
Iberian hare abundance (pellet FBII)	-177.415	460	0.0457
Mean open-air farm size (number of pigs)	0.000213	500	0.0532
Number of pigs on open-air farms	0.000209	500	0.0625
Number of pigs on open-air farms per square Km	0.1253	500	0.0949
Not selected:			
Sex, Iberian hare habitat suitability, wild rabbit abundance (pellet FBII), wild boar km abundance, wild boar spatial aggregation index (Z), wild boar abundance (dropping FBII), red deer FBII, red deer density (distance estimates), wild boar FBII by feeding site and ha, wild boar FBII by watering site and ha, annual temperature [69], mean slope, annual rainfall, annual radiation, mean altitude, sampling estate surface (Ha), type of population (open, fenced, farm), fencing, % boundary fenced, riparian habitats, irrigated cultures, non-irrigated cultures, cultivated lands, woodlands, irrigated fruit orchards, urban, tree diversity, grass cover, scrubland cover, pine woodlands, pastures, dehesa (savannah-like open oak woodlands), number of *Quercus *trees/5 m, total woodlands, tree cover, soil cover, total wood+scrublands, *Quercus *spp. >4 m/5 m, cultures (%), scrublands (%), number of waterholes, waterholes per Ha, wild boar supplemental feeding, wild boar feeding sites, wild boar feeders per Ha, deer feeding sites, goats per Ha, cattle per Ha, sheep per Ha, number of pig farms in municipality, pig farms per Km^2^, total pigs in municipality, total number of pigs in municipality per Km^2^, mean farm size (number of pigs), number of pigs on closed farms per Km^2^, closed pig farms in municipality, closed pig farms per Km^2^, mean closed farm size, pigs on closed farms, open-air pig farms in municipality, open-air pig farms per Km^2^.			

Sampling season and sampling site were included as random factors.

In the Peninsular scale model we controlled for the effect of the Bio-region by including it as categorical random variable. Factors tested are listed in Table 3.

In the smaller geographical scale model (Ciudad Real province, Bio-region 3), we restricted our analysis to wild boar sampled on 20 sites, that were well characterized regarding habitat characteristics (e.g. estate-related environmental conditions, land cover and habitat structure) and relevant wildlife management factors such as fencing, supplemental feeding, watering sites, and estimated abundance [39]. The variables tested are shown in Table 3.

Hunting season (from 2000-2001 to 2008-2009) and sampling site were included as random factors in both models.

## Results

### iELISA validation

**Figure 1:**
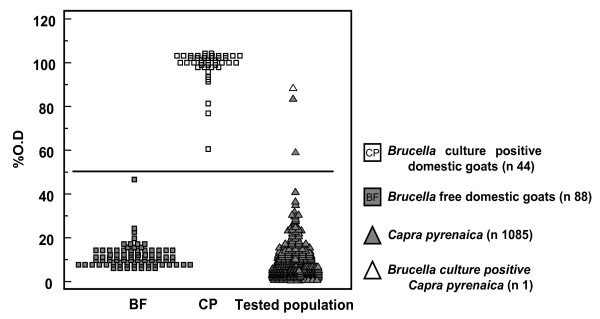
**Example of the typical distribution of optical density (% OD) results obtained by iELISA when testing the gold standard populations (from domestic goats) and its phylogenetically related Iberian wild goat (*Capra pyrenaica*) counterpart**. The horizontal line represents the cut off selected for assessing the apparent prevalence of brucellosis.

As an example of the iELISA validation procedure followed, the distribution of %OD results obtained with the gold standard populations in domestic goats and its phylogenetically related *Capra pyrenaica *counterpart is shown in Figure 1. As seen in this figure, a relatively wide range of % OD were resulting in 100% sensitivity and specificity with the gold standard populations tested, and this picture was similar when using gold standard sera from the cattle, sheep and pig populations used as reference controls. The corresponding cut-offs for the different wild animal species tested were 50% OD (for all wild ruminant species) and 40% OD (for wild boar), considering that the resulting sensitivity and specificity with the corresponding gold standard populations was always 100%.

The relative specificity of the iELISA *versus *the culture results obtained with the 50 iELISA negative wild ruminants tested (Table 2) was adequate since no positive isolations were obtained in these animals. The relative specificity versus the culture results was also adequate in wild boar, since only one *B. suis *biovar 2 strain was isolated from the cultured specimens of the 50 iELISA negative animals tested.

### Studies in wild ruminant species

Our results revealed no or only very limited antibody responses to infections by smooth *Brucella *species in Iberian wild ruminants (Table 2). Anti-*Brucella *antibodies were detected in chamois, red deer, and to a lesser extent, the Iberian wild goat. The highest apparent prevalence (0.8%) was identified in chamois, being essentially detected in the animals living in the Pyrenean Mountains, in Bio-region 2.

Altogether, the overall estimated apparent prevalence in wild ruminants was as low as 0.4% (95% CI range 0.3-0.6%), and no significant inter-species differences (Chi-square = 10.2, 6 d.f., P > 0.05) or spatial aggregation (data not shown) were evidenced. However, slightly higher apparent prevalence was observed locally. As an example, the percentage of red deer positive reactors reached maximum value of 1.9% (3 out of 158 animals tested; 95%CI 0.5-5.5) in the Garcipollera reserve (Pyrenees, Bio-region 2), and 0.8% (16 out of 1899 animals tested; range 0.5-1.4) in the Montes Universales reserve (Bio-region 4).

Only two out of the 93 animals submitted to bacteriological analyses (one from a clinical case, 42 from iELISA-positive animals, and 50 from ELISA-negative animals, Table 2) resulted in *Brucella *positive culture. One of the strains identified (*B. melitensis *biovar 1) was isolated from the clinical case, a severely ill Iberian wild goat buck found in Albacete province (Bio-region 4), and that resulted positive in the iELISA. The other strain isolated (*B. abortus *biovar 1) came from a hunter-harvested red deer stag, from Montes Universales reserve in Teruel province (Bio-region 4), and found also positive in the iELISA.

### Studies in wild boar

**Figure 2:**
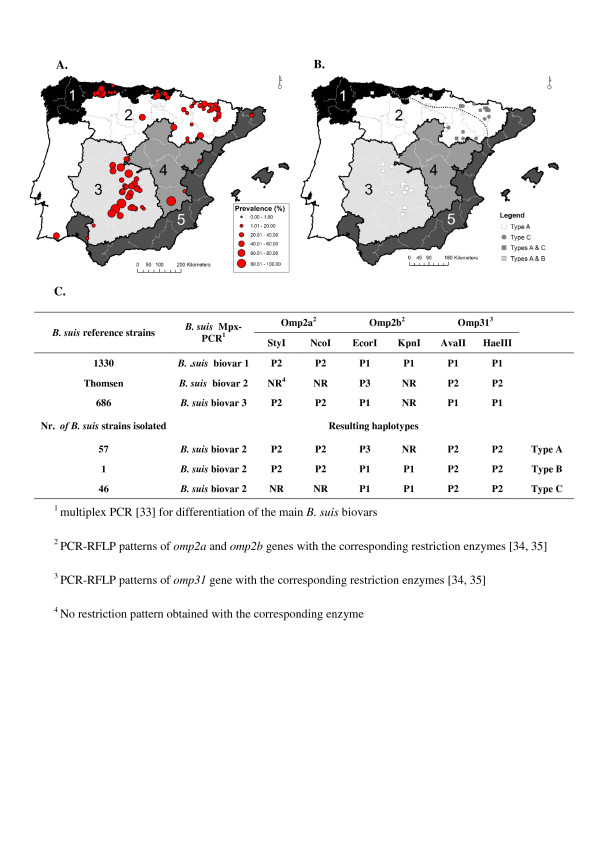
**Panel A: Apparent prevalence of brucellosis in Eurasian wild boar (*Sus scrofa*) in Bio-regions 1 to 5**. Dots are proportional to prevalence. Only data from localities with at least 10 wild boar samples are shown. Panel B: Distribution of the different haplotypes of *Brucella suis *strains isolated from wild boar. Points represent an infected population cluster rather than individual isolates; the dotted line represents the south-western distribution limit of the European brown hare (*Lepus europaeus*). Panel C: Characteristics of the *B. suis *biovar 2 haplotypes isolated when identified by PCR (Mpx-PCR) and further analysis of *omp2a*, *omp2b *and *omp31 *genes by PCR-RFLP.

In strong contrast with results found in wild ruminants, wild boar showed a high apparent prevalence of brucellosis (33%; 95%CI 31.6-34.4; see also Additional file 1), in all Bio-regions tested (Figure 2 panel A). The highest apparent prevalence (average 46% with some populations reaching over 80%) was found in Bio-region 3 (Figure 2 panel A). The remaining Bio-regions showed lower but still high values (average 26%; Figure 2 panel A). No statistically significant spatial association was found by Mantel test (Pearson r = -0.10, N = 68; P = 0.99).

A total of 539 necropsy samples from iELISA positive wild boar were submitted to bacteriological culture (Table 2). One hundred and four isolates (representing 19.3% of the animals tested) were obtained from these seropositive animals cultured, while only 1 of the 50 iELISA negative wild boar tested resulted in positive culture, being this difference statistically significant (P < 0.001). All isolates were identified as *Brucella suis*, and the multiplex PCR identified patterns consistent with those characteristic of *B. suis *biovar 2. The PCR-RFLP of *omp2a*, *omp2b *and *omp31 *genes resulted in three different *B. suis *biovar 2 haplotypes (Figure 2 panel C). Type A strains (N = 57) were found widely distributed throughout Bio-regions 1, 2 and 3, whereas type C (N = 46) and B (N = 1) strains were restricted to Bio-regions 2 and 3, respectively (Figure 2 panel B).

**Figure 3:**
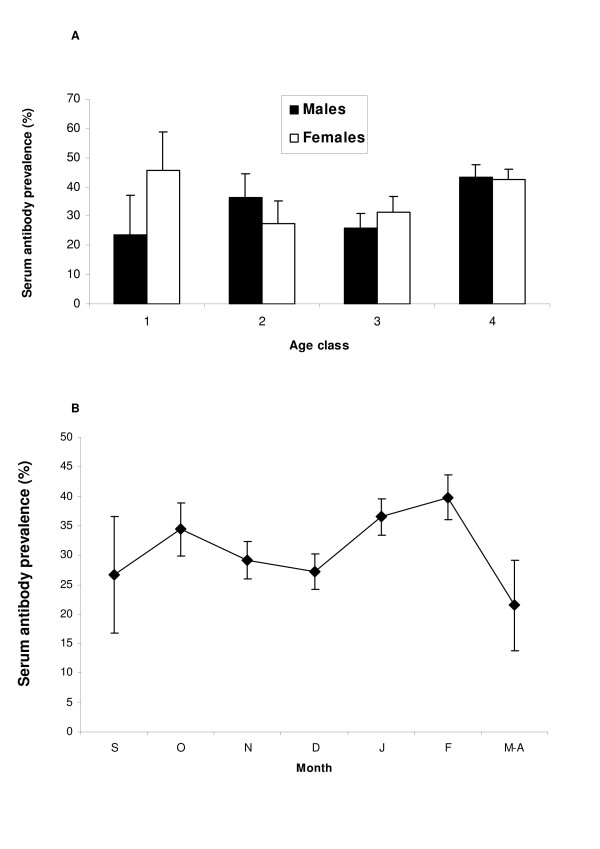
**Distribution of apparent prevalence in wild boar (*Sus scrofa*) through age and sex classes (Panel A), and sampling period (Panel B) at the Peninsular scale**.

Table 4 shows the variables included in the final large-scale model. The probability of wild boar testing positive in the iELISA was affected by age (Chi-square = 42.3, 3 d.f., P < 0.001; Figure 3 panel A), age-by-sex interaction, rainfall, Bio-region and month. By contrast, apparent prevalence was not affected by sex (males 35.8%, 95%CI 33.3-38.5; females 36.5%, 95%CI 34.0-39.0). Apparent prevalence increased during the hunting season reaching maximum levels in February (Figure 3 panel B). Apparent prevalence in wild boar also varied among Bio-regions (Chi-square = 183, 4 d.f., P < 0.001), Bio-region 3 showing almost the double of apparent prevalence than the other Bio-regions.

**Table 4:** Effects on the probability of testing positive to brucellosis at Peninsular scale.

Effect	DF	F	Pr > F
**Age**	3.1947	23.2	< 0.001
**Sex by age interaction**	4.1886	2.53	0.0390
**Rainfall**	1.186	10.7	0.0013
**Bio-region**	4.207	10.7	< 0.001
**Month**	4.1557	2.80	0.0247

DF degrees of freedom; F test statistic; Pr > F probability.

Table 5 shows the 6 variables included in the final regional-scale model. The probability of testing positive in the iELISA was affected by age-by-sex interaction, sampling month, and the number of open-air bred pigs per square Km in the sampling municipality. Fifty eight additional variables resulted not statistically significant in the first analysis and thus, not selected for the model (Table 3).

**Table 5:** Effects on the probability of testing positive to brucellosis at Regional scale.

Effect	DF	F	Pr > F
**Month**	6.373	2.39	0.0280
**Open-air pigs per square km**	1.136	3.29	0.0919
**Sex by age interaction**	5.48	4.90	0.0002

DF degrees of freedom; F test statistic; Pr > F probability.

## Discussion

We developed and validated a multi-species immunosorbent assay and applied it to determine the apparent prevalence and distribution of brucellosis in wild ungulates from the Iberian Peninsula. Our results showed that wild ruminants do not play a relevant role in the maintenance of *B. abortus *and *B. melitensis *infections. In contrast, the wild boar was identified as an important threat for *B. suis *infection.

The quality of the diagnostic methodology used is of paramount importance to assess the prevalence of wildlife diseases [28]. Due to the lack of brucellosis tests validated for wildlife species, the most recommendable approach for studies to determine brucellosis prevalence in wildlife should be based in the use of classical serological tests such as the Rose Bengal (RBT), which has been widely validated in the domestic animal species phylogenetically related with wild ungulates, and extensively used worldwide [40]. These classical tests, however, require samples of a very high quality to avoid haemolysis problems. However, gathering high quality serum samples devoid from haemolysis is frequently impossible in standard wildlife sampling procedures, particularly those based on hunted specimens. To circumvent this problem, many recent brucellosis studies in wildlife have been based on immunosorbent assays -ELISA- [17,41,42]. One of the advantages of this serological test is that the degree of haemolysis of the serum samples does not affect significantly the ELISA performance [43]. Due to the absence of specific conjugates against the immunoglobulin isotypes of the different wildlife species, indirect ELISAs have not been widely used, and most of studies have been based on the use of competitive ELISAs, which are potentially able to identify specific anti-*Brucella *antibodies in all animal species [28,44-46]. However, due to the absence of adequate gold standard sera, most studies in wildlife have been performed using the protocols (*i.e.*, serum dilution, antigen concentration, cut-off, etc) as recommended by manufacturers in domestic livestock [14,17,47], and therefore without adequate validation for the corresponding wild species tested. Moreover, the problem of the false-positive serological reactions induced by gram-negative bacteria sharing common epitopes with *Brucella *[48,49], is also an important issue to properly assess brucellosis prevalence. Hence, recent studies suggest the need for better diagnostic tools to obtain reliable results in serological studies on brucellosis in wildlife [17].

The best gold standard known in brucellosis diagnosis is the isolation of the bacteria. However, individual bacteriology is cumbersome, unpractical and very expensive to be used as the unique test to determine the prevalence of brucellosis in animal populations. Thus, the most recommendable approach is a combination of serological and bacteriological studies, such as those conducted here. We developed an iELISA using an antigen sharing the major common surface epitopes present in all smooth *Brucella *species [50,51], allowing the diagnosis of infections induced by *B. abortus, B. melitensis *and *B. suis*. The lack of availability of polyclonal or monoclonal antibodies raised to detect specifically the immunoglobulin isotypes of wildlife species was overcome by using protein G as a conjugate. This reagent has been reported suitable in wildlife for detecting antibodies to *Brucella *[52,53] and other pathogens [54,55]. Due to the absence of gold standard sera from culture positive and brucellosis free wild animals, we validated our iELISA using gold standard sera from the closest phylogenetically related domestic species. The adequate relative sensitivity of the iELISA with respect to the bacteriological status of the animals was confirmed in wild boar, in which the number of strains isolated from seropositive animals was relatively high (Table 2), being comparable to those obtained in similar studies conducted in the EU [52].

The success for bacteriological isolation depends on the quality of the samples cultured. Unfortunately, in our study it was not always possible to obtain necropsy samples of proper quality, which probably decreased the final sensitivity of the bacteriological methods applied. This can explain the relatively high number of samples from iELISA positive animals that resulted in negative culture. Moreover, the relative specificity of the iELISA *versus *culture results was also adequate since only one out of the 50 iELISA negative animals tested yielded a positive culture. However, this iELISA negative serum from an infected wild boar could also be due to a recent *B. suis *infection in which antibodies of the IgG isotypes (the only ones detected by protein G) had not yet been produced, or simply, as a consequence of a human error in sampling or identification.

The relative sensitivity of the iELISA developed could not be properly assessed in wild ruminants due to the low apparent prevalence figures detected and, accordingly, the low number of iELISA positive samples cultured (Table 2). The only two animals in which field *Brucella *strains were isolated resulted positive in the iELISA. Finally, no brucellae were isolated from the 50 iELISA negative wild ruminants tested, this result supporting the adequate relative specificity of the serological test developed. Therefore, this iELISA should be considered as adequate enough for detecting *Brucella *antibodies in the wild species studied.

At least for the species with large sampling sizes (Table 2), it can be concluded that wild ruminants are not a significant potential source of *B. abortus *and *B. melitensis *infections for livestock in the Iberian Peninsula. However, data on species with a limited sample size, such as Barbary sheep (N = 8) and mouflon (N = 75), are not enough to support that general conclusion. The finding of the *B. melitensis *infected Iberian wild goat in a locality with no active sampling stresses, however, the importance of setting up passive wildlife surveillance networks.

The small variations in the geographical distribution of seropositive wild ruminants can reflect sampling biases rather than real differences in apparent prevalence. However, the relatively high apparent prevalence found in some areas could be also related with the high prevalence of brucellosis in domestic species reared in extensive breeding systems. As an example, the percentage of red deer and chamois positive reactors reached maximum values in some areas of the Pyrenees (Bio-region 2), and red deer in the Montes Universales reserve (Bio-region 4), that were coincident with some brucellosis outbreaks taking place in domestic sheep and cattle in these mountain areas during the 2002 and 2004 seasons (Gobierno de Aragón, Annual Animal Health Report, unpublished data).

Current knowledge on *B. abortus *epidemiology in the Yellowstone area strongly suggests that artificial management including crowding and supplemental feeding influences the dynamics of wildlife brucellosis [13]. The very low apparent prevalence of brucellosis in Iberian wild ruminants may be explained by a couple of non-mutually excluding hypotheses. First, the relatively low overall prevalence of brucellosis in domestic ruminants in Spain makes the transmission to wildlife highly improbable, despite the existence of important risk factors such as overabundance [27]. Second, artificial feeding in southern Spain takes place mostly in summer, once the lambing/calving season is over. Thus, abortions occurring at winter feeding sites as in elk in the Yellowstone area [13], are unlikely. This is consistent with recent results on the effects of management on elk behaviour and brucellosis transmission [56].

In strong contrast with the situation in wild ruminants, the wild boar population was found seriously affected by *B. suis *biovar 2 infection. The general apparent prevalence figures found herein (Table 2) were similar to those indicated in other European reports [10,14-17,57]. However, apparent prevalence close to 100% was recorded locally (Figure 2). Bio-region 3, the area where game is more intensively managed through fencing, feeding and translocation, was the region with the highest apparent prevalence (Figure 2 panel A). This Bio-region concentrates practically the whole Iberian censuses of domestic Iberian pigs reared in fully out door breeding systems.

The absence of sex effects on brucellosis apparent prevalence in wild boar (Table 3) was not surprising, since similar results have been found also in other diseases [39,58]. However, we found at both geographical scales a significant effect of the sex-by-age interaction on the apparent prevalence of brucellosis (Table 3). This effect can be explained by sex and age related differences in wild boar behaviour [59]. While females live in matriarchal groups, adult males live solitary and only contact with these matriarchal groups during the mating season [60]. Apparent prevalence observed among adult wild boar was higher than that found in younger age classes, as expected by the higher participation in reproduction by adults [61].

In wild boar, positivity to several other infectious agents has been linked with density, spatial aggregation or artificial management (e.g. Aujeszky's disease [61,62]; Bovine tuberculosis [39]; Porcine circovirus type 2 [58]). However, no relationship between apparent prevalence and wild boar management or density risk factors has been evidenced in this study. There is no clear explanation for this finding, and further research is needed to better identify the factors modulating *B. suis *infection.

Several authors have suggested that spillover from wild boar and European hares to domestic pigs could be a frequent event, and the explanation of the re-emergence of brucellosis due to *B. suis *biovar 2 in outdoor reared pigs in EU countries [63,64]. Historical contact between free ranging Iberian domestic pigs and wild boar could have boosted wild boar infection with *B. suis *biovar 2 in the Iberian Peninsula. As indicated above, Bio-region 3 is the Spanish region with more open-air bred domestic pigs [26], and in which the apparent prevalence figures in wild boar were maximal (Figure 2). In the small scale study carried out in this Bio-region 3, a positive relationship between apparent prevalence in wild boar and the density of open air bred Iberian pigs was evidenced (Table 5). This may contribute to explain the important prevalence of brucellosis reported in Iberian pig farms in the last years in Spain [24,65]. Accordingly, having in consideration the close genetic characteristics of the strains isolated in Spain [25], our study confirms that domestic Iberian pigs reared outdoor and wild boar share the same brucellosis infection due to *B. suis *biovar 2. Three out of the five wild boar estates showing the highest apparent prevalence were fully open and sharing pastures with free-ranging domestic pigs.

In an attempt to further characterise the *B. suis *biovar 2 infection in wild boar, a DNA-based study was applied to all strains isolated (Figure 2 panel C). None of the three *B. suis *haplotypes identified (A, B and C; Figure 2 panel C) were coincident with the molecular patterns characteristic of the *B. suis *biovar 2 Thomsem reference strain (Figure 2 panel C). However, these three haplotypes were consistent with those previously identified in domestic pigs and wild boar in Spain, Portugal and other European countries [24,66]. The *B. suis *strains previously isolated from pigs and wild boar in the Iberian Peninsula corresponded exclusively to both A and B haplotypes, and with a neat predominance of type A (32 strains) *versus *type B (10 strains) [24]. In agreement with this, the type A strains isolated (57 strains) were largely predominant on type B (only one strain isolated) in our study (Figure 2, panel C), and were widely distributed in Bio-regions 1, 2 and 3 (Figure 2, panel B). The haplotype C had never been reported previously in the Iberian Peninsula, but it has been reported in domestic pigs from France and Croatia and also in wild boar from France, Italy and Switzerland [24]. Surprisingly, this haplotype C was found in similar proportions as haplotype A (Figure 2, panel C). However, and interestingly, this haplotype was restricted exclusively to Bio-region 2 (Figure 2, panel B). This can explain the absence of previous reporting of this particular haplotype in Spain since none of the papers published were dealing with strains isolated from this Bio-region.

Altogether, it can be concluded that *B. suis *biovar 2 strains isolated from Iberian wild boar are spatially structured. This structuring was conserved despite frequent translocations taking place for hunting purposes [67].

In contrast with the situation reported in France [68], wild boar were capable to maintain *B. suis *biovar 2 infection independently of the existence of European brown hares. Interestingly, the unique *B. suis *biovar 2 strain isolated from European brown hare in Spain [25] was showing a molecular pattern different from the three haplotypes identified in this study in wild boar (J.M Blasco, unpublished results). This hare strain was showing also different restriction patterns from those identified in the *B. suis *biovar 2 Thomsen reference strain and other *B. suis *biovar 2 strains isolated from hares in France, which show common patterns with those identified in wild boar (B. Garin-Bastuji, personal communication). This suggests that at least in Spain, the *B. suis *biovar 2 haplotypes infecting European brown hares and wild boar may be different. However, this must be confirmed in further studies using larger numbers of animals. The possible role of the Iberian hare (*Lepus granatensis*) in *B. suis *biovar 2 epidemiology is currently unknown. No isolation of *B. suis *biovar 2 has been reported in Iberian hares but no adequate studies are available. Suitability of Iberian hare habitat, meaning open, flat, sparsely-forested Mediterranean agrosystems, was selected in the first step of the analysis, but not in the final model. Its weak link with wild boar apparent prevalence may be due to a correlation between Iberian hare habitat suitability and Bio-region 3. A similar explanation can be given for the inclusion of rainfall in the large-scale model, having in consideration that rainfall is more abundant in the North (e.g. Bio-region 1) than in Bio-region 3 (Table 1).

Data provided herein suggest that *B. suis *biovar 2 infection can be maintained in wild boar in an independent epidemiological cycle to that taking place in domestic pigs. The period of the year (month of sampling) was a significant factor affecting apparent prevalence (Tables 4, 5), suggesting that the reproductive season may influence brucellosis spreading among wild boar. An alternative explanation could be related with differences in host-specific behaviour, for example regarding carrion consumption from gut piles during the hunting season (October to February).

## Conclusions

In summary, we conclude that free-living wild ruminants are not a significant brucellosis reservoir in the Iberian Peninsula but conversely, wild boar is an important threat regarding *B. suis *biovar 2 infection. This represents an important hazard particularly for the Iberian pig population reared in out door breeding systems, but the entry of the disease in the highly intensified pig industry should not be disregarded. This situation could become of great concern if brucellosis control programs in domestic pigs are envisaged.

## Competing interests

The authors declare that they have no competing interests.

## Authors' contributions

Conceived and designed the study: CG, JB, JF, JV, MB. Participated in sampling and field work: MB, MA, JV, PA, AO, FR, DF, MP. Carried out the laboratory work: PM, MR, DM, CM, MoB. Analyzed the data: MB, JV, PA, CG. Drafted the manuscript: PM, MB, JB, CG. All authors read and approved the final manuscript.

## Pre-publication history

The pre-publication history for this paper can be accessed here:

http://www.biomedcentral.com/1471-2334/10/46/prepub

## Supplementary Material

Additional file 1 **Detailed wild boar *Brucella *antibody seroprevalence by Bio-region**. Data shows sample size, number of ELISA positive samples, and serum antibody prevalence of wild boar from the Iberian Peninsula. Click here for file
