# Supplementary material for: Spatial distribution and risk factors of Brucellosis in Iberian wild ungulates
Source: BMC Infect Dis. 2010 Mar 5;10:46. doi: 10.1186/1471-2334-10-46 (PMC2841660; doi:10.1186/1471-2334-10-46)
Supplement: Additional file 1 — Detailed wild boar Brucella antibody seroprevalence by Bio-region. Data shows sample size, number of ELISA positive samples, and serum antibody prevalence of wild boar from the Iberian Peninsula. [file 1471-2334-10-46-S1.DOC]

**Additional file 1.-** Sample size (1), number of ELISA positive samples (2), and serum antibody prevalence (3) of wild boar from the Iberian Peninsula.

| **1.- SAMPLE SIZE BY BIO-REGION, AGE, AND SEX** | | | | | | |  |  |  |  |  |  |  |  |  |  |  |  |  |  |  |  |  |  |
| --- | --- | --- | --- | --- | --- | --- | --- | --- | --- | --- | --- | --- | --- | --- | --- | --- | --- | --- | --- | --- | --- | --- | --- | --- |
| Bioregion | Age Class | |  |  |  |  |  |  |  |  |  |  |  |  |  |  |  |  |  |  |  |  |  | TOTAL |
|  | 1 |  |  |  | 2 |  |  |  | 3 |  |  |  | 4 |  |  |  | Age unknown | |  |  | All ages | |  |  |
|  | Male | Female | Unknown | Total | Male | Female | Unknown | Total | Male | Female | Unknown | Total | Male | Female | Unknown | Total | Male | Female | Unknown | Total | Male | Female | Unknown | |
| 1 | 0 | 4 | 2 | 6 | 13 | 9 | 0 | 22 | 32 | 33 | 1 | 66 | 61 | 67 | 2 | 130 | 42 | 47 | 345 | 434 | 148 | 160 | 350 | 658 |
| 2 | 10 | 6 | 2 | 18 | 22 | 29 | 3 | 54 | 125 | 128 | 2 | 255 | 249 | 233 | 13 | 495 | 107 | 104 | 887 | 1098 | 513 | 500 | 907 | 1920 |
| 3 | 39 | 45 | 34 | 118 | 102 | 104 | 11 | 217 | 139 | 140 | 1 | 280 | 251 | 332 | 17 | 600 | 16 | 27 | 241 | 284 | 547 | 648 | 304 | 1499 |
| 4 | 0 | 1 | 0 | 1 | 7 | 12 | 3 | 22 | 7 | 8 | 0 | 15 | 14 | 33 | 6 | 53 | 12 | 12 | 17 | 41 | 40 | 66 | 26 | 132 |
| 5 | 2 | 1 | 0 | 3 | 7 | 2 | 1 | 10 | 7 | 4 | 0 | 11 | 22 | 21 | 0 | 43 | 0 | 0 | 178 | 178 | 38 | 28 | 179 | 245 |
| TOTAL | 51 | 57 | 38 | 146 | 151 | 156 | 18 | 325 | 310 | 313 | 4 | 627 | 597 | 686 | 38 | 1321 | 177 | 190 | 1668 | 2035 | 1286 | 1402 | 1766 | 4454 |
|  |  |  |  |  |  |  |  |  |  |  |  |  |  |  |  |  |  |  |  |  |  |  |  |  |
| **2.- ELISA (+) SAMPLES** | | |  |  |  |  |  |  |  |  |  |  |  |  |  |  |  |  |  |  |  |  |  |  |
| Bioregion | Age Class | |  |  |  |  |  |  |  |  |  |  |  |  |  |  |  |  |  |  |  |  |  | TOTAL |
|  | 1 |  |  |  | 2 |  |  |  | 3 |  |  |  | 4 |  |  |  | Age unknown | |  |  | All ages | |  |  |
|  | Male | Female | Unknown | Total | Male | Female | Unknown | Total | Male | Female | Unknown | Total | Male | Female | Unknown | Total | Male | Female | Unknown | Total | Male | Female | Unknown | |
| 1 |  | 4 | 1 | 5 | 7 | 1 |  | 8 | 11 | 14 | 0 | 25 | 23 | 32 | 0 | 55 | 9 | 11 | 68 | 88 | 50 | 62 | 69 | 181 |
| 2 | 0 | 0 | 0 | 0 | 2 | 2 | 0 | 4 | 10 | 10 | 0 | 20 | 84 | 64 | 4 | 152 | 34 | 24 | 262 | 320 | 130 | 100 | 266 | 496 |
| 3 | 12 | 22 | 8 | 42 | 42 | 38 | 2 | 82 | 58 | 70 | 0 | 128 | 146 | 181 | 7 | 334 | 10 | 16 | 83 | 109 | 268 | 327 | 100 | 695 |
| 4 |  | 0 |  | 0 | 4 | 5 | 0 | 9 | 1 | 3 |  | 4 | 4 | 13 | 0 | 17 | 2 | 0 | 2 | 4 | 11 | 21 | 2 | 34 |
| 5 | 0 | 0 |  | 0 | 0 | 0 | 0 | 0 | 0 | 1 |  | 1 | 2 | 1 |  | 3 |  |  | 60 | 60 | 2 | 2 | 60 | 64 |
| TOTAL | 12 | 26 | 9 | 47 | 55 | 46 | 2 | 103 | 80 | 98 | 0 | 178 | 259 | 291 | 11 | 561 | 55 | 51 | 475 | 581 | 461 | 512 | 497 | 1470 |
|  |  |  |  |  |  |  |  |  |  |  |  |  |  |  |  |  |  |  |  |  |  |  |  |  |
| **3.- SERUM ANTIBODY PREVALENCE** | | | | |  |  |  |  |  |  |  |  |  |  |  |  |  |  |  |  |  |  |  |  |
| Bioregion | Age Class | |  |  |  |  |  |  |  |  |  |  |  |  |  |  |  |  |  |  |  |  |  | MEAN |
|  | 1 |  |  |  | 2 |  |  |  | 3 |  |  |  | 4 |  |  |  | Age unknown | |  |  | All ages | |  |  |
|  | Male | Female | Unknown | Mean | Male | Female | Unknown | Mean | Male | Female | Unknown | Mean | Male | Female | Unknown | Mean | Male | Female | Unknown | Mean | Male | Female | Unknown | |
| 1 |  | 100,00 | 50,00 | 83,33 | 53,85 | 11,11 |  | 36,36 | 34,38 | 42,42 | 0,00 | 37,88 | 37,70 | 47,76 | 0,00 | 42,31 | 21,43 | 23,40 | 19,71 | 20,28 | 33,78 | 38,75 | 19,71 | 27,51 |
| 2 | 0,00 | 0,00 | 0,00 | 0,00 | 9,09 | 6,90 | 0,00 | 7,41 | 8,00 | 7,81 | 0,00 | 7,84 | 33,73 | 27,47 | 30,77 | 30,71 | 31,78 | 23,08 | 29,54 | 29,14 | 25,34 | 20,00 | 29,33 | 25,83 |
| 3 | 30,77 | 48,89 | 23,53 | 35,59 | 41,18 | 36,54 | 18,18 | 37,79 | 41,73 | 50,00 | 0,00 | 45,71 | 58,17 | 54,52 | 41,18 | 55,67 | 62,50 | 59,26 | 34,44 | 38,38 | 48,99 | 50,46 | 32,89 | 46,36 |
| 4 |  | 0,00 |  | 0,00 | 57,14 | 41,67 | 0,00 | 40,91 | 14,29 | 37,50 |  | 26,67 | 28,57 | 39,39 | 0,00 | 32,08 | 16,67 | 0,00 | 11,76 | 9,76 | 27,50 | 31,82 | 7,69 | 25,76 |
| 5 | 0,00 | 0,00 |  | 0,00 | 0,00 | 0,00 | 0,00 | 0,00 | 0,00 | 25,00 |  | 9,09 | 9,09 | 4,76 |  | 6,98 |  |  | 33,71 | 33,71 | 5,26 | 7,14 | 33,52 | 26,12 |
| MEAN | 23,53 | 45,61 | 23,68 | 32,19 | 36,42 | 29,49 | 11,11 | 31,69 | 25,81 | 31,31 | 0,00 | 28,39 | 43,38 | 42,42 | 28,95 | 42,47 | 31,07 | 26,84 | 28,48 | 28,55 | 35,85 | 36,52 | 28,14 | 33,00 |
